# Case Report: Rothmund-Thomson syndrome type 2 in Ecuador: clinical and molecular insights into a recurrent *RECQL4* variant

**DOI:** 10.3389/fped.2026.1695356

**Published:** 2026-02-09

**Authors:** Martina Isabella Armas Samaniego, Mateo Briones Vasquez, Mel Mariño Zambrano, Vanessa I. Romero, Karen Melo, Juan Carlos Pozo-Palacios

**Affiliations:** 1 Escuela de Biotecnología, Colegio de Ciencias Biológicas y Ambientales, Universidad San Francisco de Quito, Quito, Ecuador; 2 Escuela de Medicina, Colegio de Ciencias de la Salud, Universidad San Francisco de Quito, Quito, Ecuador; 3 Instituto de Microbiología, Colegio de Ciencias Biológicas y Ambientales, Universidad San Francisco de Quito, Quito, Ecuador; 4 Servicio de Oncología Pediátrica, Hospital Carlos Andrade Marin, Quito, Ecuador; 5 Escuela de Medicina, Universidad de Cuenca, Cuenca, Ecuador

**Keywords:** clinical genetics, Ecuador, molecular diagnosis, rare diseases, *RECQL4*, Rothmund-Thomson syndrome type 2

## Abstract

This case report presents two Ecuadorian patients with Rothmund-Thomson syndrome type 2 (RTS2), an autosomal recessive disorder, who share a *RECQL4* variant previously identified in another Ecuadorian patient, supporting the recurrent presence of this variant in the Ecuador population. Additionally, in the Case 2 patient with a suspected compound heterozygosity, a second pathogenic variant was identified that had not been previously reported in Ecuador. These findings underscore the importance of molecular diagnosis for accurate classification of RTS2, informed risk assessment, and improved clinical care, particularly in underrepresented populations.

## Introduction

Rothmund-Thomson syndrome type 2 (RTS2, OMIM:268400) is a genetic disorder characterized by early-onset poikiloderma (HP:001029) -skin rash with pigment changes (HP:0025300), atrophy (HP:0003202) and telangiectasias (HP:0100585)-, short stature (HP:0004322), and sparse or absent scalp hair (HP:0002209; HP:0002293), eyebrows (HP:0002223; HP:0045075), and eyelashes (HP:0200102). Additional features include dental (HP:0000164), nail (HP:0001597), and skeletal anomalies (HP:0011842), with a high risk of osteosarcoma (HP:0002669) in childhood and skin squamous cell carcinoma (HP:0002860) later in life (1, 2).

RTS2 is an autosomal recessive disorder caused by pathogenic variants in the *RECQL4* gene, which encodes the RecQ-like helicase 4. This helicase plays a central role in maintaining genomic stability by unwinding double-stranded DNA, regulating chromosome segregation, and participating in critical processes such as DNA replication, homologous recombination, and repair of DNA damage. *RECQL4* also contributes to the preservation of telomere and mitochondrial DNA integrity (3). Its expression is most prominent in the thymus and testis.

In this case report, we describe two unrelated patients diagnosed with RTS2, detailing their clinical features and molecular findings. Both patients carry the same pathogenic variants (Case 1-homozygous and Case 2 heterozygous), which was previously reported in an Ecuadorian patient with RTS2 who was adopted into an Italian family. Additionally, we identify a second variant in Case 2 that has not been previously described in clinical literature.

## Patient description

### Case 1

Case 1 involves a 4-year-old male who presented to the outpatient clinic with progressive dermatologic manifestations. At 6 months of age, he developed generalized erythema (HP:0007432), which gradually evolved into poikiloderma (HP:0001029) predominantly affecting the limbs, abdomen, and facial region. Additional clinical features included brittle hair (HP:0002299) and hypoplastic nails (HP:0001792). On physical examination, the patient exhibited short stature (−2 SD) (HP:0004322) and a varus deformity of the lower limbs (HP:0002814). There was no reported family history of genetic disorders or parental consanguinity. Laboratory tests revealed chronic neutropenia (1,090 /mm³) (HP:041252). Skeletal radiographs showed limb deviation (HP:0040069) and mild radial hypoplasia (HP:002984). Genetic testing, performed through a commercial laboratory using the Invitae Multi-Cancer Panel, identified a homozygous likely pathogenic variant in the *RECQL4* gene: c.3236G>T (p.Ser1079Ile; rs2130657545). The variants were analyzed according to the ACMG/AMP international guidelines and classified as likely pathogenic based on the criteria PM2, PM5, and PP3. This classification indicates that the variant has an extremely low allele frequency in the gnomAD population (PM2)—being absent in the Admixed American subgroup—, is a different amino acid change as a known pathogenic variant (PM5) and is a missense variant that the computational prediction tool unanimously support a deleterious effect on the gene (PP3). Variant information was corroborated using standardized databases, including gnomAD, ClinVar, PubMed, LOVD, dbSNP, NCBI Genome, RefSeqGene, and Franklin Genoox. In ClinVar, the variant is reported as likely pathogenic for RTS2. According to gnomAD, it has a global allele frequency of 6.225 × 10⁻⁷ and is not reported in the Admixed American population (frequency 0.00), supporting its classification as a rare disease–associated variant. No images are available for this patient due to privacy restrictions.

### Case 2

**Figure 1:**
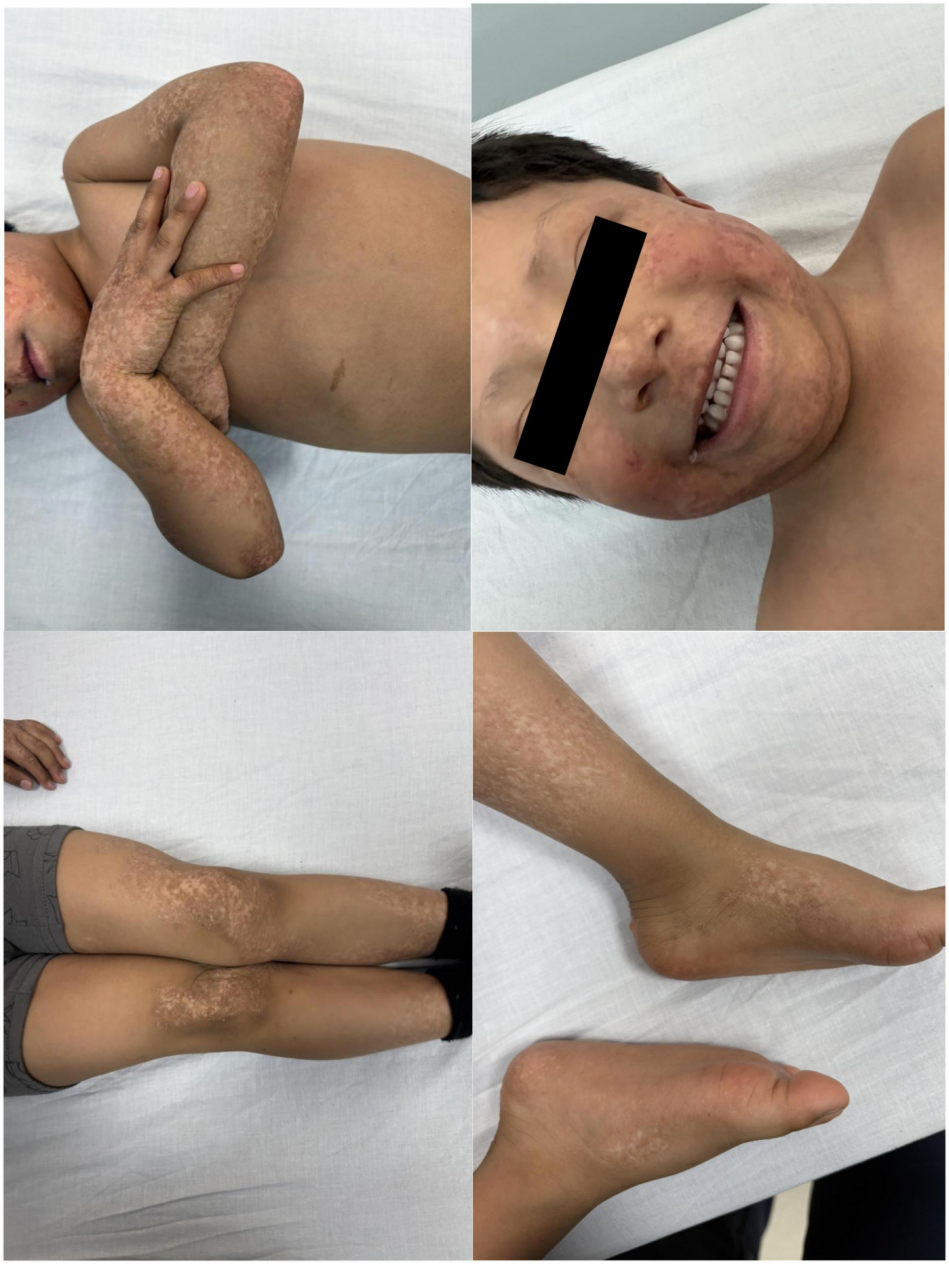
Images onf Case 2 patient showing widespread poikiloderma across the body. Patient showing widespread poikiloderma across the body, including the face (cheeks, nose, and jaw), arms and hands, legs, knees, and feet (including soles). No signs of dental abnormalities are observed. Sparse eyebrows are also noted.

Case 2 describes a 3-year-old boy who was seen at the outpatient clinic due to worsening skin-related symptoms. At 6 months of age, he developed a bilateral malar rash (HP:0025300). By 15 months, erythematous lesions appeared on the upper and lower extremities, sparing the trunk and buttocks (HP:0025475). By age 2, the lesions had spread diffusely across the body (Figure 1). A skin biopsy revealed basal layer vascularization (HP:0002671), hyperkeratosis (HP:0000962), and mild epidermal acanthosis (HP:0025092), supporting a clinical diagnosis of RTS. There was no reported family history of genetic disorders or parental consanguinity. At 2 years and 7 months, the patient showed signs of delayed language development (HP:0000750), difficulties with conceptual understanding (HP:5200116), and reduced muscle strength (HP:0001324). Additional findings included sparse eyebrows and eyelashes (HP:0045075; HP:0200102), as well as white hair near the scalp (HP:0011364).

Whole-exome sequencing (WES) was performed through a commercial laboratory and identified two variants in the *RECQL4* gene. Variants interpretation was conducted according to ACMG/AMP international guidelines, and classifications were corroborated using standardized databases, including gnomAD, ClinVar, PubMed, LOVD, dbSNP, NCBI Genome, RefSeqGene, and Franklin Genoox. Two *RECQL4* variants were identified in Case 2. However, parental segregation analysis was not available; therefore, the allelic phase (cis/trans) could not be confirmed, and compound heterozygosity remains inferred based on the clinical phenotype and autosomal recessive inheritance pattern. The first was a heterozygous likely pathogenic variant, c.3236G>T (p.Ser1079Ile; rs2130657545), also detected in Case 1. This variant is classified as likely pathogenic in ClinVar and is absent from the Admixed American population in gnomAD. The second was a heterozygous pathogenic variant, c.1243C>T (p.Gln415*; rs2130712164), which introduces a premature stop codon at amino acid position 415 in exon 6. This nonsense variation is expected to result in early termination of translation and loss of normal protein function. According to ACMG/AMP criteria, this variant meets PVS1 (predicted loss of function), PM2 (extremely low allele frequency in gnomAD, absent in the Admixed American subgroup), and PM3 (detected in trans in a recessive disorder in a compound heterozygous state), supporting its classification as pathogenic. The variant has a global gnomAD frequency of 6.202 × 10⁻⁷ and has not been previously reported in ClinVar or the published literature, indicating that it represents a novel pathogenic variant associated with the phenotype observed in these patients. Therefore, this patient presents a compound heterozygous state, supported by parental segregation analysis demonstrating that the two variants are in trans. Both parents are clinically unaffected and report no disease-related symptoms, consistent with an autosomal recessive inheritance pattern in which each parent carries a single pathogenic variant. Confirmation could not be access due to limitations in access to parents’ sample and lack of resources.

## Discussion

Prior to our report, only three cases of RTS had been documented in the Ecuadorian population. Two of these were described by Romero-Flórez in 2012 and were based solely on clinical findings, as molecular diagnostics were not accessible within Ecuador's public health system at the time. The first case involved a 7-year-old girl diagnosed at 6 months with poikiloderma (HP:0001029) and bilateral agenesis of the thumbs (HP:0009601). Skin biopsy revealed histological features consistent with RTS, including hyperkeratosis (HP:0000962), epidermal hyperplasia with alternating areas of basal hyperpigmentation and flattening (HP:0025092), loss of rete ridges (HP:0025117), vacuolization of the basal layer (HP:0002671), and pigment incontinence (HP:0034572). She was later diagnosed with osteosarcoma (HP:0002669) and died (HP:0003819) in 2012 due to treatment-related complications. There is no information available about history of genetic disorders in the family or parental consanguinity The second case, a 15-year-old girl, also developed poikiloderma (HP:0001029) starting at 6 months of age. She presented with sparse scalp hair (HP:0002209), absence of eyebrows and eyelashes (HP:0002223; HP:0200102), and bilateral absence of thumbs (HP:0009601). She subsequently developed osteosarcoma (HP:0002669) with pulmonary metastases and died (HP:0003819) in 2012 (4). There is no information available about history of genetic disorders in the family or parental consanguinity.

The third reported case, described by Colombo et al. in 2018, involved an Ecuadorian boy adopted by an Italian family. He developed poikiloderma (HP:0001029) at 6 months, beginning on the cheeks and progressing to the arms and forearms, a pattern consistent with that seen in our current patients. Additional findings included onychodystrophy (HP:0008394) and keratoconus (HP:0000563). There is no information available about history of genetic disorders in the family or parental consanguinity. Unlike the earlier cases, this patient underwent molecular testing in Italy, which revealed a homozygous *RECQL4* variant, c.3236G>T (p.Ser1079Ile). This same variant was identified in homozygosity in our Case 1 and in compound heterozygosity in Case 2, suggesting a possible recurrent or founder variant in the Ecuadorian population (Table 1) (5).

**Table 1:** Summary of Ecuadorian RTS2 cases from literature and this report.

RTS common findings		Case 1	Case 2	Romero-Flórez Patient 1	Romero-Flórez Patient 2	Colombo et al. Patient
Birth/death		2015	2022	2005–2012	1997–2012	1995
Sex		Masculine	Masculine	Femenine	Femenine	Masculine
Growth	Height	Within normal	Within normal	Short	Short	<3rd percentile
	Weight	Minus 2SD	Within normal	Low	Low	25th–50th percentile
	Milestones	Normal	Delayed motor and verbal	Normal	Not reported	Not reported
Skin	Poikiloderma	Starting at 6 months	Starting at 6 months	Starting at 6 months	+ (Age not reported)	Starting at 6 months
	Facial erythema	+	+	+	+	+
	Hyperkeratosis	+	+	+	+	+
	Sparse scalp hair, eyelashes, and/or eyebrows	+	+	+	+	+
Mouth	Rudimentary or hypoplastic teeth	−	−	+	Not reported	Not reported
	Microdontia	−	−	+	Not reported	Not reported
	Increased incidence of caries	−	+	Not reported	Not reported	Not reported
Hands	Dysplastic or hypoplastic nails	+	−	+	+	−
	Rudimentary or hypoplasic thumbs	+	−	+	+	−
Eyes	Cataracts	−	−	−	Not reported	Keratoconus
Skeletal	Radius or ulna defects	Radius hypoplasia	−	Bilateral absence of radius and curved ulnar	Radius hypoplasia	-
	Bone density	Not performed	Not performed	Not reported	Not reported	Low bone density
Cancer	Osteosarcoma	−	−	+	+	−
Skin biopsy		Not performed	Hyperkeratosis, basal vacuolization, pigment incontinence, dyskeratotic cells, a lymphocytic infiltrate with eosinophils, melanophages, and ectatic vessels.	Hyperkeratosis, lentiginous epidermal hyperplasia with focal basal hyperpigmentation and flattening, basal vacuolization, pigment incontinence, and a scant lymphocytic infiltrate at the interface	Not reported	Not reported
Hematologic alterations		Neutropenia	Not performed	Not reported	Not reported	−
*RECQL4* variation		c.3236G>T (p.Ser1079Ile)/c.3236G>T (p.Ser1079Ile)	c.3236G>T (p.Ser1079Ile)/c.1243C>T (p.Gln415*)	Not reported	Not reported	c.3236G>T (p.Ser1079Ile)/c.3236G>T (p.Ser1079Ile)

+ = Present, − = Absent.

Among the five Ecuadorian RTS patients, cutaneous manifestations were universally present and formed the core diagnostic feature. However, variability in skeletal anomalies (HP:0005775), dental defects (HP:0000164), neurologic development (HP:0012758), and cancer susceptibility highlights the phenotypic heterogeneity of RTS, even within a shared geographic or genetic context.

*RECQL4* is a RecQ helicase that contributes to genomic stability and plays a role in the initiation of DNA replication (6). The c.3236G>T (p.Ser1079Ile) variant is located within the Zn^2+^-binding domain of *RECQL4*, a conserved region composed of two antiparallel *α*-helices and four cysteine residues that coordinate a zinc ion (6). This domain plays a critical role in the base excision repair (BER) pathway, which repairs DNA base damage caused by methylation, oxidation, and related modifications (6). Notably, this region mediates the interaction between *RECQL4* and *PARP1*, a key DNA damage sensor that recruits and stabilizes *RECQL4* at sites of oxidative damage (7). The p.Ser1079Ile substitution may interfere with this interaction, potentially impairing BER efficiency by disrupting repair complex formation and DNA processing (8). However, data regarding this variant are scarce; thus, additional functional studies are required to confirm this proposed mechanism. The variant is absent from the Admixed American population in the Genome Aggregation Database, supporting the possibility of a population-specific variant in Ecuador. Less than 1% of genomic data in databases represents Latin American individuals, which may explain the limited representation of variants from this region. None of the reported Ecuadorian patients are related, yet all originate from the same area in the northern highlands, supporting the possibility that this variant is recurrent in the broader Ecuadorian population.

Its presence in all three genetically analyzed patients—along with its recurrence in an unrelated Ecuadorian case reported abroad—reinforces the hypothesis of a founder mutation. While the two earlier cases reported by Romero-Flórez lacked genetic testing, confirming the presence of this variant in those individuals would provide stronger evidence for this hypothesis and help clarify potential ethnic or regional inheritance patterns. This same variant was identified in homozygosity in our Case 1 and in compound heterozygosity in Case 2, supporting that c.3236G>T (p.Ser1079Ile) represents a recurrent *RECQL4* variant observed in Ecuadorian patients (Table 1) (5).

Its presence in all three genetically analyzed Ecuadorian patients—together with its recurrence in an unrelated Ecuadorian case reported abroad—supports that c.3236G>T (p.Ser1079Ile) is a recurrent *RECQL4* variant in Ecuador. Confirming the presence of this variant in the earlier clinically diagnosed cases reported by Romero-Flórez would further strengthen this observation and help define its frequency and geographic distribution within the country.

Living at altitudes above 2,000 meters requires consideration of relevant clinical factors. The dysfunction of the *RECQL4* gene, which is responsible for DNA repair and the maintenance of genomic stability, should be considered in populations that are chronically exposed to such strong environmental stressors (9). Chronic hypoxia increases oxidative stress, thereby contributing to greater genomic damage (10, 11). Additionally, ultraviolet (UV) radiation increases by approximately 10%–12% for every 1,000 meters of elevation (12), which may exacerbate the characteristic skin manifestations of the syndrome. The use of sunscreen should be considered a crucial part of the treatment for patients diagnosed with RTS2. Unfortunately, medical professionals often overlook this aspect, which reflects a broader issue within the Ecuadorian healthcare context.

Reports of RTS2 in Latin America remain scarce and are largely limited to Brazil, Mexico, and Puerto Rico (13). In Brazil, a cohort study reported biallelic *RECQL4* variants in 6 out of 12 evaluated patients (14). None of the variants identified in Brazilian patients overlap with those found in Ecuadorian cases. The most frequently reported Brazilian variants include the intronic c.588−2214A>G, exon 4 deletion, exon 15 deletion, c.1711dup (p.Ile571Asnfs*27), and c.143T>C (p.Leu48Pro) (14). In Mexico, a case reported in 1967 described an affected woman treated at Eagleville Hospital; however, no molecular characterization was available (13, 15). More recently, in 2022, two RTS2 cases were reported in Mexico, but only one underwent genetic sequencing, revealing a homozygous *RECQL4* variant without detailed variant information (16). In Puerto Rico, a case involving a 6-year-old girl born to non-consanguineous parents was reported in 1987, though no genetic analysis was performed (17). In 2025, a case involving an African American/Puerto Rican child identified compound heterozygous pathogenic *RECQL4* variants (p.Ser523fs and p.Ala805_Arg807del), neither of which correspond to the variants observed in Ecuadorian patients (18).

The Ecuadorian variants identified in this study—p.Ser1079Ile and p.Gln415*—have not been previously described in the literature, underscoring a lack of scientific reports analyzing these variants. Although genetic studies of RTS2 in Latin America are limited, the available evidence suggests that the variants identified in Ecuador may be population-specific, highlighting the need for further molecular characterization in underrepresented populations.

Limited access to molecular diagnostics in Ecuador remains a significant barrier to early diagnosis and proper genetic counseling. A limitation of this study is the absence of segregation testing for Case 2. Although the presence of two *RECQL4* variants supports RTS2 in the appropriate phenotypic context, we could not confirm whether the variants are in trans due to the unavailability of parental samples and resource constraints. Another limitation of this report is the lack of Ecuadorian or regional reference frequency data for RECQL4 variants, which prevents direct comparison with local population allele frequencies. Larger studies in Ecuador and Latin America will be required to define the regional frequency of these variants. The absence of molecular data in earlier reports reflects systemic healthcare limitations. Nevertheless, our study contributes valuable data to the characterization of RTS in Ecuador and underscores the urgent need to improve access to genetic testing and precision diagnostics in underserved populations.

## Conclusion

This case report describes two new Ecuadorian patients with RTS2, both carrying the c.3236G>T (p.Ser1079Ile) variant in *RECQL4*, supporting that this is a recurrent variant observed in Ecuadorian patients. One patient also harbored a second pathogenic variant not previously reported in Ecuador, c.1243C>T (p.Gln415*). These findings highlight the importance of molecular diagnosis for accurate classification, risk assessment, and improved clinical management of RTS2, particularly in underserved regions.

## Data Availability

The raw data supporting the conclusions of this article will be made available by the authors, without undue reservation.
